# Effects of Achieving Sustained Virologic Response after Direct-Acting Antiviral Agents on Long-Term Liver Fibrosis in Diabetics vs. in Non-Diabetic Patients with Chronic Hepatitis C Infection

**DOI:** 10.3390/biomedicines10092093

**Published:** 2022-08-26

**Authors:** Marian-Sorin Popescu, Dan-Mihai Firu, Vlad Pădureanu, Cristina Maria Mărginean, Radu Mitruț, Andreea Letitia Arsene, Dragoș Nicolae Mărgăritescu, Daniela Calina, Anca Oana Docea, Paul Mitruț

**Affiliations:** 1 Department of Medical Semiology, University of Medicine and Pharmacy of Craiova, 200349 Craiova, Romania; 2 Department of Cardiology, University and Emergency Hospital, 050098 Bucharest, Romania; 3 Department of Microbiology, Carol Davila University of Medicine and Pharmacy, 020021 Bucharest, Romania; 4 Department of Surgery, University of Medicine and Pharmacy of Craiova, 200349 Craiova, Romania; 5 Department of Clinical Pharmacy, University of Medicine and Pharmacy of Craiova, 200349 Craiova, Romania; 6 Department of Toxicology, University of Medicine and Pharmacy of Craiova, 200349 Craiova, Romania

**Keywords:** hepatitis C viral infection, type 2 diabetes mellitus, direct-acting agents, insulin resistance, FibroMax

## Abstract

Because of the prevalence of HCV worldwide as well as its undiagnosed population due to a lack of screening, HCV can be considered a modern pandemic disease. In 2016, the World Health Organization (WHO) set goals for HCV’s elimination that included a 65 percent reduction in mortality and an 80 percent reduction in newly infected cases by 2030. This study is a follow-up evaluation of 80 patients who received interferon-free treatment with direct-acting agents (DAA) for chronic HCV infection between the second half of 2017 and the end of 2018. They were assessed using a FibroMax test prior to DAA administration. Two pills/day of Ombitasvir 12.5 mg/Paritaprevir 75 mg/Ritonavir 50 mg and two pills/day of Dasabuvir 250 mg were given to the patients for 8 weeks. After treatment, all 80 patients in this study achieved an SVR (sustained virologic response), and the FibroMax test was performed three years later. Our study found that successfully treating HCV infection can play a significant role in reducing fibrosis in T2DM patients. In comparison to those of ActiTest and SteatoTest, FibroMax scores showed a significantly greater reduction in T2DM patients than in treatment-naive patients.

## 1. Introduction

Hepatitis C virus (HCV) infection encompasses approximately 170 million cases worldwide with an increasing trend. As of 2021, 422 million diabetes cases were reported by the World Health Organization (WHO), and that number grows yearly. HCV can be considered a modern pandemic disease due to the prevalence of HCV worldwide together with its undiagnosed population due to a lack of screening. In 2016, the World Health Organization (WHO) set goals for HCV’s elimination that included a 65% reduction in mortality and an 80% reduction in newly infected cases by 2030 [1]. The invention of direct-acting agents (DAAs) was a significant step toward achieving this goal [2]. One of the limitations on the success of modern DAAs is their high cost, which is responsible for their limited use, as well as some drug-resistant mutations that present a new challenge to be overcome [3]. Another limitation is a late diagnosis, since a significant number of patients are unaware of the infection. A late diagnosis occurs when a patient is already presenting severe symptoms, such as advanced fibrosis or cirrhosis [4]. Despite successful treatment and obtaining an SVR (sustained virologic response), which can help decrease the risk of HCC (hepatocellular carcinoma), the positive effects of DAAs are decreased in those with advanced fibrosis or cirrhosis [5].

Hepatitis C and diabetes are two diseases of our time that affect a significant amount of the global population. The chronic form of hepatitis C is a risk factor for diabetes, and patients with diabetes are more likely to develop a more complicated form of hepatitis C [6]. Up to one-third of people with chronic hepatitis C also have type 2 diabetes mellitus [7,8]. In many studies throughout the years, a two-way association between the two diseases has been found.

A considerable number of studies showed that there is an increased risk for chronic HCV-infected patients to develop type 2 diabetes mellitus (T2DM) [9]. Studies on the HCV population have concluded that one in three patients with HCV will develop at least one of the possible extrahepatic manifestations, such as glomerulonephritis, porphyria cutanea tarda, mixed cryoglobulinemia, or T2DM [10]. Some developed this conclusion based on direct viral involvement or on liver damage caused by HCV infection [11]. While some studies consider chronic HCV infection to be a risk factor for developing T2DM [12], few studies supported this association if HCV infection did not progress to liver dysfunction. Researchers observed a higher incidence of T2DM in the case of patients with elevated alanine aminotransferase (ALT) [13]. To support the two-way association, some studies reported T2DM to be a predisposing factor for HCV infection [14,15]. Among the complications of HCV infection, insulin resistance (IR) and T2DM are now more frequently reported. The mechanism involved in these extrahepatic manifestations is related to chronic inflammation induced by a HCV infection that disturbs the central role of the liver in glucose homeostasis, insulin resistance (IR), and impaired glucose tolerance [16]. Post-mortem studies revealed that HCV can also replicate in tissues other than those of the liver and pancreas, particularly pancreatic acinar cells and epithelial cells of the pancreatic duct [17]. The structural and non-structural HCV proteins seem to be the main cause of IR and oxidative stress induction at the cellular level [18]. The core protein of HCV is implicated in the upregulation of insulin receptor substrate-1 (IRS-1) phosphorylation. The phosphorylated IRS-1 activates phosphatidylinositol 3-kinase (PI3K) that is implicated in the insulin’s metabolic effects, along with Akt dysregulation [19]. In addition to IR impairment, a disturbance in Akt/PKB activation is another step in glucose uptake inhibition that can explain the increased incidence of T2DM in HCV-infected patients [20].

The association between cirrhosis and glucose intolerance has been identified in many studies that associated cirrhotic patients with impaired glucose tolerance [21]. Since the 1994 study with Allison’s group, epidemiologic studies have linked T2DM with HCV-induced cirrhosis [22]. The pathogenic role of HCV infection in the development of T2DM was further supported by statistics showing that HCV infection preceded a T2DM diagnosis in approximately 73% of cases [23]. 

Approximately one in three chronic HCV patients are estimated to have T2DM [24]. While over 30 studies showed that HCV poses a significantly high risk for T2DM [25], a few studies indicated that the findings may be due to variations in the HCV genotype, patient ethnicity, the severity of liver disease, or other possible variables [24,26]. A NHANES study revealed no correlation with diabetes [27] and found only elevated ALT and gamma-glutamyl transpeptidase (GGT) to be associated with HCV infection [28]. However, the data tend to indicate that the risk for T2DM is between two and ten times higher in HCV infection than when compared with other liver diseases [29]. Researchers concluded that there is a significant risk for T2DM among chronic HCV-infected patients and that the association is not simply linked to cirrhosis or coincidental [30]. 

This study investigated both the changes in liver fibrosis as assessed by a FibroMax score taken after HCV clearance was obtained for T2DM and non-T2DM patients and also the impact of viral clearance on glucose levels and insulin resistance. 

## 2. Materials and Methods

### 2.1. Study Design

This study represented a follow-up evaluation of 80 patients (40 with TD2M and 40 without TD2M) that underwent DAA treatment for chronic HCV infection. As part of the diagnostic protocol, the HCV genotype was established in all patients as genotype 1b, the most common genotype in southeastern European countries.

Each patient received 2 pills/day of Ombitasvir 12.5 mg/Paritaprevir 75 mg/Ritonavir 50 mg (Viekirax) and 2 pills/day of Dasabuvir 250 mg (Exviera) for 8 weeks. The DAAs were produced by AbbVie in the USA. These patients were referred to the Renasterea Private Clinic and the Department of Internal Medicine at the Emergency County Hospital Craiova between the second half of 2017 and the end of 2018. At that time, they were evaluated with the help of a FibroMax test before DAA administration. All 80 of the patients in this study obtained an SVR after treatment and at the time of the follow-up; the FibroMax test was done 3 years after the treatment ended and an SVR was obtained. The subjects of the study were selected from those who were newly initiated (59 patients, referred to “treatment-naive” patients) and those that had initially undergone interferon-based treatment regimens (21 patients, referred to as “treatment-experienced patients”) and had failed to obtain an SVR at 24 weeks. An SVR was defined as the absence of HCV RNA after 12 weeks of DAA therapy. All patients included in the study were evaluated annually on their HCV viral load, liver function biomarkers, and alpha-fetoprotein levels along with an abdominal ultrasound imaging evaluation of the liver architecture. The HCV RNA was absent at all evaluation time points. 

Prior to being included in the study, all participants gave written consent and were informed of the procedure involved and that they could withdraw at any time. The protocol for this study was constructed to follow the ethical guidelines listed in the Declaration of Helsinki and was approved by the ethics committee of the University of Medicine and Pharmacy of Craiova. 

### 2.2. Molecular Determination of HCV RNA and Genotype

The HCV RNA levels in each patient’s blood were determined by real-time PCR using the automated instrument COBAS Amplicore (ROCHE diagnostics, Indianapolis, IN, USA). Genotyping was performed using a TruGene 5′NC HCV Genotyping kit (Siemens Healthcare Diagnostics Division, Tarrytown, NY, USA).

### 2.3. Diagnosis of T2DM Patients

The diagnosis of T2DM was made prior to the DAA treatment and was based on fasting blood glucose levels (above 126 mg/dl for T2DM) and a glycated haemoglobin test (HbA1c) (above 6.5% for T2DM). 

### 2.4. FibroMax Test

A FibroMax test is a non-invasive method of diagnosing liver disease based on blood samples by evaluating biomarkers from serum. FibroMax tests are used in the clinical practices of infectious disease specialists, gastroenterologists, and haematologists worldwide. Five different combinations of tests (FibroTest, ActiTest, SteatoTest, NashTest, and AshTest) are included under the name FibroMax. Each of the five parameters was obtained with the help of calculations on the BioPredictive test (Biopredictive, France) based on 10 biochemical assays: Alpha2-macroglobulin (g/L), haptoglobin (g/L), apolipoprotein A1 (APOA1)(mg/dL), GGT (gamma-glutamyl transpeptidase) (IU/L), total bilirubin (TB) (mg/dL), ALT (alanine aminotransferase) (IU/L), AST (aspartate aminotransferase) (IU/L), fasting glucose (mg/dL), total cholesterol (TC) (mg/dL), and triglycerides (mg/dL) [29]. The results of these assays were adjusted to the patient’s age, gender, weight (kg), and height (meters) for the calculation of the FibroMax tests. The 10 parameters were obtained with the analyzer Architect C8000 (ABBOTT Corporation, Chicago, IL, USA) with closed-system reagents from the same company using immunonephelometric methods (alpha2-macroglobulin, haptoglobin, and apolipoprotein A1), colourimetric methods (total bilirubin), enzymatic methods (ALT, AST, and GGT), and enzymatic-colorimetric methods (cholesterol, triglycerides, and fasting glucose). 

Fibrosis was staged in correspondence with the scoring system F0–4. The ActiTest was staged as grades A0–3 corresponding to the section of the scoring system assessing viral necroinflammatory activity. The SteatoTest measured the steatosis grade in hepatocytes in the range of S0–3. The NashTest evaluated the level of necroinflammatory activity caused by the metabolic condition and was measured as N0–2. The AshTest evaluated the level of necroinflammatory activity due to alcohol consumption. 

The body mass index (BMI) was calculated for each subject, taking into account the age, weight, and height of the subject.

### 2.5. Statistical Analysis

The obtained data were stored in Microsoft Excel files (Microsoft Corp., Redmond, WA, USA), and the statistics were calculated in the software STATA (StataCorp LLC, College Station, TX, USA). We investigated the relationship between T2DM and liver fibrosis in previously HCV-infected patients and the effect of an SVR obtained after 3 years for those parameters. The numerical data were reported as the median of the values and interquartile range (IQ) (p25–p75). The graphical representations and calculation of the regression coefficients were performed with STATA. For the complex statistical tests, we used the Mann–Whitney–Wilcoxon test, Kruskal–Wallis test when data were not normally distributed, or Student’s *t*-test when the distribution of values was gaussian. For the discrete data, we used the chi-squared test with Cramer’s V as a measure of association (effect size) of the strength of the relationship between the diabetes state and the liver fibrosis class. 

## 3. Results

The group consisted of 80 patients divided into two equal cohorts (40 patients per group), T2DM patients and non-T2DM patients that previously had FibroMax test scores evaluated before interferon-free treatment. These patients had a follow-up FibroMax test evaluation done after the viral load test had confirmed that they retained an SVR 3 years after treatment. 

As the distribution shows in both groups, there were almost twice as many female participants as there were male participants in both groups. A slight difference was found in the habitat. A quarter of the patients had previously undergone interferon treatment without obtaining an SVR before this study, with seven times more treatment-naive patients in the non-TD2M group compared with the TD2M group. The liver function parameters ALT, AST, and TB were similar in both groups at baseline. We observed a significant difference in the lipid profile parameters between the baseline values of TC, but with triglycerides and APOA1. The BMI was also similar in both groups (Table 1).

### 3.1. Analysis of Raw FibroMax Scores

**Figure 1:**
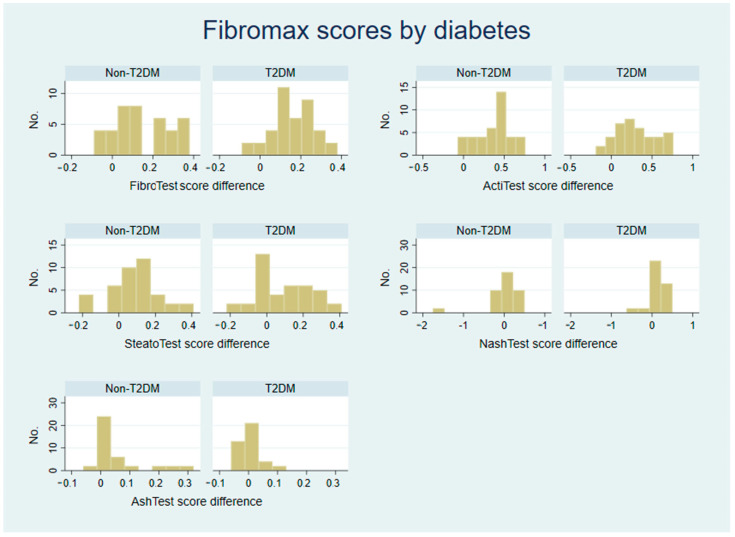
Distribution of changes in the FibroMax test scores after DAA treatment in non-T2DM patients versus in T2DM patients.

As shown by the results of the follow-up FibroMax tests after 3 years, the average values for the FibroTest decreased in both categories, proving that an SVR has a significant impact on fibrosis scores. The non-T2DM group showed an average median decrease of 0.13, slightly lower than the 0.16 decrease in T2DM patients, and had a lower median value of 0.71, compared with 0.74 for T2DM patients. The non-T2DM patients showed slightly better results in the case of the ActiTest scores, a median decrease of 0.41 compared with just 0.22 for the T2DM group, as well as slightly better SteatoTest scores, 0.09 compared with 0.08 for the T2DM group. Since the diabetic patients had better results in the NashTest with an interquartile range of 0.00–0.25, most of them had a decrease in the score, compared with −0.17–0.14 for the non-T2DM group. In the AshTest score, the diabetic group showed no change in test results since treatment ended (Table 2 and Table 3 and Figure 1).

Based on the ActiTest results, a successful treatment led to the overall improvement of inflammation scores except in a few patients. The patients showed significant improvement. On average, the difference between the initial scores and post-treatment revealed a median of a 66% decrease for the ActiTest score, while in the case of diabetics, this value was halved, reaching 31% (Table 2 and Table 3 and Figure 1).

When we compared the fasting glucose levels, we observed a statistically significant difference in the case of T2DM patients. In our study group, the diabetics significantly improved the management of their glucose levels, which dropped by 27 mg/dL for these patients. Among the non-T2DM group, we also observed that even if the overall decrease was small (2 mg/dL), the levels actually increased by 9 mg/dL for some patients, possibly indicating a possible prediabetic state; whereas for others, there was a decrease of 11 mg/dL, but the difference was not statistically significant (Table 4).

The results from the follow-up SteatoTests revealed that achieving an SVR did not have a statistical impact on the degree of steatosis in both groups. However, the T2DM group recorded a significantly lower improvement in this test median. Among the non-T2DM group, 75% of the patients decreased their SteatoTest score, compared with only 50% of patients in the T2DM group. The association between T2DM and HCV infection also presented a higher baseline degree of steatosis (Table 2 and Table 3 and Figure 1). 

The results of the follow-up revealed that, in the case of T2DM patients, treatment for HCV led to a decrease in the NashTest score of 90% of the patients, while only 70% of non-T2DM patients showed an improvement on this test. The test results also showed that 24% of non-T2DM values had increased after an SVR was achieved, and 83.3% of them advanced to the next class on the scale. Conversely, only 8% of T2DM patients presented higher values on the follow-up, and all patients in this category progressed to the next class on the scale (Table 2 and Table 3 and Figure 1). 

When considering the AshTest results, we observed a reduction of more than 60% in the non-TD2M group after treatment with DAAs, compared with no reduction in the TD2M group. However, when analyzing individual patients, we observed that 75% of the patients from the T2DM group achieved an improvement in the AshTest class, compared with 90% of the patients in the non-TD2M group. Remarkably, 50% of patients from both groups showed no improvement in this score after treatment with DAAs (Table 2 and Table 3 and Figure 1).

### 3.2. Analysis of FibroTest Classes

**Figure 2:**
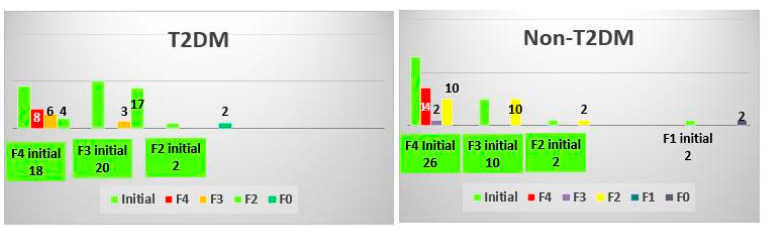
Changes in the FibroTest class after treatment in the T2DM and non-TDM2 groups.

Except for a few patients that had slightly elevated results compared with their initial levels, all other participants showed improvements. The FibroTest scores of T2DM and non-T2DM patients revealed that some of them even dropped two classes on the fibrosis scale (25% in the non-TD2M group and 35% in the TD2M group). The biggest improvements were noticed among the most advanced stages of fibrosis, specifically in class 3 and 4 in both T2DM and non-T2DM patients. To a lesser extent, the T2DM patients with class 1 and 2 fibrosis also showed improvements in the FibroTest score, still managing to drop to an inferior stage (Figure 2). The Cramer’s V effect size was 0.253, and the *p*-value was 0.076. 

In our study groups, the biggest benefit of obtaining an SVR seemed to be noticeable for the advanced stages of the disease (F4 fibrosis). Successful interferon-free treatment led to 55% of T2DM patients decreasing the severity level of their fibrosis, compared with 46% of non-T2DM patients. 

### 3.3. Analysis of ActiTest Classes

**Figure 3:**
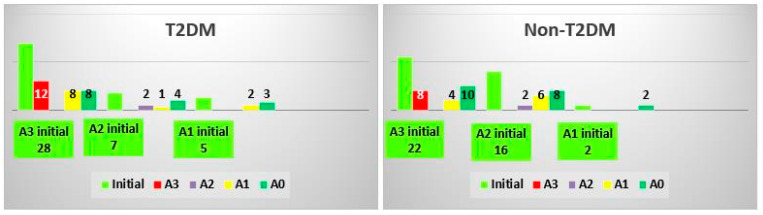
Changes in the ActiTest class after treatment in the T2DM and non-TDM2 groups.

In both groups, 35% of patients did not regress to an inferior ActiTest class. In the case of T2DM patients, 57% regressed from the advanced class, compared with 63% in the non-T2DM group (Figure 3). The Cramer’s V effect size was 0.151, and the *p*-value was 0.612. 

### 3.4. Analysis of SteatoTest Classes

**Figure 4:**
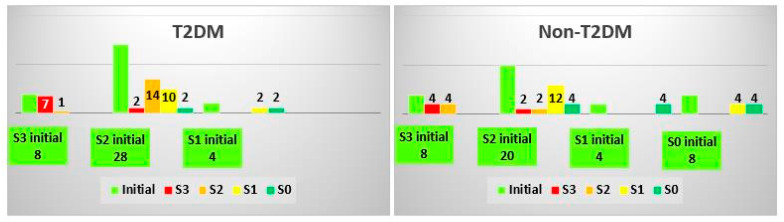
Changes in the SteatoTest class after treatment in the T2DM and non-TDM2 groups.

Unlike the other tests, the degree of steatosis in some patients showed increased levels and advanced to higher classes on the SteatoTest scale after a follow-up evaluation. In the TD2M group, 5% of patients had higher results in the follow-up test, and in the non-TD2M group, 15% had progressed to the next level of steatosis (Figure 4). The Cramer’s V effect size was 0.340, and the *p*-value was 0.026. 

### 3.5. Analysis of NashTest Classes

**Figure 5:**
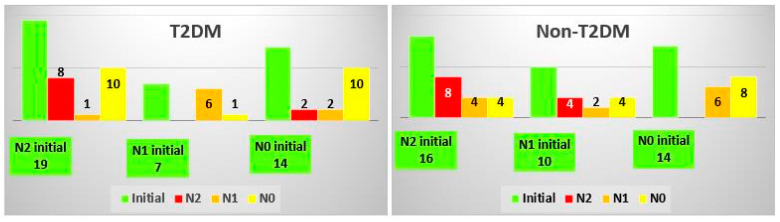
Changes in the NashTest class after treatment in the T2DM and non-TDM2 groups.

One interesting fact revealed by our tests was that the same patients who had higher SteatoTest results had higher NashTest results as well, except for a couple of T2DM and non-T2DM patients who presented elevated results in all tests (Figure 5). In the TD2M group, only 28% of patients with an N0 class escalated to N1 or N2. In the non-TD2M group, 40% of patients with an N1 class escalated to N2, and 28% of patients with an N0 class escalated to an N1 class. The Cramer’s V effect size was 0.374, and the *p*-value was 0.024. 

### 3.6. Analysis of AshTest Classes

**Figure 6:**
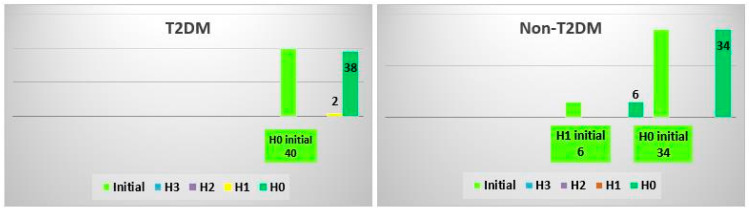
Changes in the AshTest class after treatment in the T2DM and non-TDM2 groups.

In the case of AshTest, no significant change was noticed for T2DM patients. However, there was an improvement in the non-T2DM group, where all H1 patients regressed to an H0 class after an SVR was achieved (Figure 6). The Cramer’s V effect size was 0.321, and the *p*-value was 0.016. 

### 3.7. Analysis of the Impact of Previous Hepatitis Treatment on the Effect of Current Treatment with DAAs on Liver Fibrosis

In treatment-experienced patients, the DAAs treatment significantly reduced liver fibrosis in T2DM patients as measured through FibroTest (Cramer’s V = 0.645, *p*= 0.013), but not as measured through ActiTest, SteatoTest, NashTest, or AshTest. In contrast, in treatment-naive patients, most FibroMax scores showed a significantly greater reduction in diabetic patients vs. in non-T2DM patients (ActiTest Cramer’s V = 0.393, *p* = 0.028; SteatoTest Cramer’s V= 0.406, *p* = 0.021; NashTest Cramer’s V = 0.493, *p* < 0.001; AshTest Cramer’s V = −0.354, *p* = 0.025).

### 3.8. Regression Analysis

The multivariate regression analysis (Table 5) showed a great influence of baseline value on the post-treatment value for all FibroMax components except NashTest. The T2DM impacted the improvement of NashTest and AshTest without reaching statistical significance, and the treatment-naive status of each patient significantly improved the SteatoTest score. A high BMI (body mass index) led to an increase in FibroTest, ActiTest, and SteatoTest scores.

## 4. Discussion

In the absence of cirrhosis or antiviral therapy, chronic HCV infection is associated with T2DM [31], indicating that it is not the stage of liver disease alone that has an impact on the development of diabetes. The presences of fibrosis and cirrhosis were isolated as independent risk factors that play a role in the development of T2DM, according to a few studies [32]. Various studies showed higher rates of T2DM in HCV cirrhosis than in other causes of cirrhosis, including alcohol, cholestatic disease, and HBV [25]. These results suggest a direct diabetogenic effect of HCV beyond the damage to the liver. Studies also showed that T2DM rates increased even more based on higher grades of hepatic fibrosis, steatosis, or cirrhosis [33]. One study showed that the prevalence of T2DM increased with the rise of the fibrosis score of HCV patients with an OR of 3,83 [34]. Conversely, T2DM itself was recently demonstrated to accelerate the progression of fibrosis and cirrhosis, solidifying the bidirectional association between insulin resistance and chronic HCV infection [35]. 

While numerous studies have proven that HCV infection constitutes a risk factor for T2DM, patients with insulin resistance (IR) and HCV were shown to have significantly worse clinical outcomes. The two-way association exemplifies the role of insulin resistance in chronic HCV infection. IR was associated with increased liver fibrosis, higher rates of HCC, and a reduced response to antiviral therapy [36]. An SVR by DAAs led to an important reduction in the risk of HCC [37]. Our study showed that, after an SVR was obtained, some T2DM patients had lower blood glucose levels, similar to results observed after HCV virus eradication with interferon-based therapies [38]. Some of the non-T2DM patients presented higher-than-normal glucose levels, possibly hinting at a prediabetic state. This prediabetic state could be partially attributed to the impact HCV had on the patient. The same effects were observed in previous studies, where the chronic liver inflammation associated with an HCV infection led to an increase in the prediabetic stage, diabetes, and steatosis [39].

Several factors increase the risk of liver fibrosis and subsequent cirrhosis; these include a longer duration of infection, an older age at the time of exposure, a co-infection with other viruses, alcohol consumption, and being a man, to list a few of them [40]. Our study emphasized that the T2DM population presented a higher average fibrosis degree, which coincides with other studies that demonstrated IR to be an independent predictor of fibrosis in HCV infection [41]. The study of Hsu et al. found a strong association between the severity of fibrosis and diabetes [42]. Even if their findings are uniform, however, some studies failed to show this correlation and concluded that post-load IR can be a valid predictor of fibrosis progression [43]. Overall, the majority of studies support the notion that diabetes and IR play a role in liver fibrosis and cirrhosis. 

Hepatic steatosis is the accumulation of lipids (fatty acids and triglycerides) in the liver and inside the liver cells, and while it can occur as a consequence of alcohol consumption (alcoholic hepatic steatosis), it also accompanies obesity/metabolic syndrome [44]. Hepatic steatosis in patients with chronic HCV can be a direct result of HCV or IR and T2DM. 

The eradication of HCV RNA with genotype 3 was found to be associated with the resolution of hepatic steatosis [45]. However, in other HCV genotypes where steatosis is an established component of the metabolic syndrome, achieving an SVR did not appear to resolve hepatic fat [46]. It is well-known that the majority of patients with HCV infections in Romania have genotype 1b [47]. Our study observed the same in the case of the non-T2DM group, where the results of the follow-up FibroMax showed no significant variation. These results were consistent with those observed in IR and T2DM (Table 1), suggesting that the eradication of HCV cannot reverse the signs of the metabolic syndrome once they have been established [48,49]. Another important aspect observed was that before treatment, the patients with both diabetes and an HCV infection had a higher average steatosis level, as shown by the SteatoTest (Table 1). 

In the case of the NashTest, the follow-up results revealed that non-T2DM patients were less likely to decrease on the NASH scale. While T2DM patients presented an average score decrease of 0.11 points, the non-TD2M patients’ score decreased by only 0.06 points. In the case of the ActiTest results, T2DM patients presented a higher median decrease (we used median as the distribution was not normal) score, compared with those of non-T2DM patients, proving that diabetes enhances the inflammation in HCV infection. For our study, the changes seen in the AshTest were decreased only for the non-T2DM group (all patients with an H1 score regressed to H0), which could be attributed to a change in the patient’s lifestyle by removing alcohol from their diet. The absence of any variation in the results of the T2DM group requires further testing and a larger cohort of patients to draw any conclusion. Numerous studies show that chronic HCV infection leads to or worsens IR and T2DM and contributes to coronary and cerebral vascular disease as well as chronic kidney disease. Our analysis showed that some T2DM patients experienced better glycemic control. The data show that the successful treatment of HCV can reduce the risk of developing IR and T2DM, improve IR and glycemic control, and thus reduce the risk of morbidity and mortality in patients with metabolic syndrome significantly [49]. 

Moreover, diabetes mellitus is known to be associated with a high risk of atherosclerosis and cardiovascular disease. Therefore, the extrahepatic effects of HCV eradication by DAAs recently demonstrated in some studies are extremely important in the diabetic population. In fact, following an SVR by DAAs, a significant reduction in major cardiovascular events was recently observed [50,51]. Moreover, the eradication of HCV infection by DAAs, leading to a reduction in chronic inflammatory status, resulted in a lowering in incidence of type 2 diabetes as well [52].

The regression analysis showed that almost all scores, except those of the NashTest, improved after DAA treatment, which is similar to the results of previous studies [53]. Even if it did not reach statistical significance, diabetes led to an improvement in the NashTest score, reflecting a decrease in hepatosteatosis after viral clearance, linked to the improvement of glycemic control as previously reported [7]. In patients with a higher BMI, the fibrosis scores improved less after viral clearance, as BMI was associated with a reduced effect of antiviral therapy on liver fibrosis, similar to the results of the study by Delgado-Borrego et al. [54].

One limitation of this study is the relatively low number of patients per TD2M and non-TD2M groups, which limits the power to detect statistically significant differences between groups. Additionally, because the patients were selected from only two medical centres, there is a possibility of bias. Another limitation is the relatively low number of “treatment-experienced” patients (21 patients), which limits the analysis of this group. Future studies should include other ethnicities as well as multiple checkups spread through several years to better understand how diabetes influences cirrhosis. 

Information on the characteristics of the diabetic patients studied (TD2M duration, glycemic control expressed in HbA1c, and antidiabetic therapy) was not fully collected as the study was retrospective and those were not in the scope of the study at the time of DAA treatment. All the above parameters can influence the evolution of HCV-related chronic liver disease in the diabetic population. Therefore, future studies should include diabetes parameters in order to discover the benefits of HCV eradication on TD2M progression. From the multitude of lipidic profile parameters, we chose only the total cholesterol, triglycerides, and APOA1, as these are relevant for the evaluation of liver fibrosis using a FibroMax test. The correlation between HCV eradication and blood levels of ApoB, ApoE, and VLDL should be analyzed in future studies, as these are important subclasses of lipids in the context of diabetes.

## 5. Conclusions

Our study showed that the successful treatment of an HCV infection can play a significant role in reducing fibrosis for treatment-experienced T2DM patients. For treatment-naive patients, we noticed that FibroMax scores showed a significantly greater reduction in diabetic patients, specifically in regards to the ActiTest and SteatoTest. 

## Figures and Tables

**Table 1:** General characteristics of the patients included in the study.

		Non-T2DM Group	T2DM Group
Variable	Category		
Sex (no., %)	Female	26 (65.00%)	23 (57.50%)
	Male	14 (35.00%)	17 (42.50%)
Habitat (no., %)	Rural	22 (55.00%)	22 (55.00%)
	Urban	18 (45.00%)	18 (45.00%)
Prior treatment (no., %)	Yes	16 (40.00%)	5 (12.50%) *
	No	24 (60.00%)	35 (87.50%) *
HCV genotype	1b	40 (100%)	40 (100%)
Age (years) (median, IQ range)		65 (58–71)	65 (59–70)
ALT (IU/L) (median, IQ range)		85 (61.5–131)	82 (59–108)
AST (IU/L) (median, IQ range)		68 (49–92)	72 (54–88)
TB (mg/dL) (median, IQ range)		0.78 (0.62–1.03)	0.67 (0.54–0.88)
BMI		24.70 (22.83–28.73)	25.85 (24.65–29.00)
TC (mg/dL) (median, IQ range)		144 (128–174)	172 (141–264) *
Triglycerides (mg/dL) (median, IQ range)		104 (64–138)	104 (73–126)
APOA1 (mg/dL) (median, IQ range)		1.39 (1.23–1.56)	1.47 (1.35–1.63)

Note: * *p* < 0.05 for the TD2M group compared with the non-TD2M group.

**Table 2:** FibroMax parameters in non-T2DM patients vs. in T2DM patients both before DAA treatment (initial) and 3 years after DAA treatment (final).

Parameter	Statistic	FibroTest		ActiTest		SteatoTest		NashTest		AshTest	
		Initial	Final	Initial	Final	Initial	Final	Initial	Final	Initial	Final
Non-T2DM	Median	0.74	0.54	0.62	0.16	0.57	0.42	0.50	0.50	0.04	0.02
	IQ range	0.67–0.84	0.42–0.78	0.50–0.78	0.11–0.39	0.36–0.63	0.25–0.75	0.25–0.75	0.25–0.70	0.01–0.11	0.01–0.04
T2DM	Median	0.71	0.54	0.70	0.24	0.56	0.52	0.50	0.25	0.02	0.03
	IQ range	0.64–0.85	0.46–0.68	0.53–0.74	0.12–0.62	0.49–0.65	0.40–0.66	0.25–0.75	0.25–0.59	0.01–0.04	0.01–0.04
*p* value		0.326	0.672	0.500	0.189	0.699	0.086	0.503	0.150	0.024 *	0.270

Note: * *p* < 0.05 when comparing the non-T2DM group with the TD2M group; IQ range: interquartile range.

**Table 3:** Variation in FibroMax parameters after DAA treatment in non-T2DM patients vs. in T2DM patients.

Parameter	Statistic	FibroTest Score Decrease	ActiTest Score Decrease	SteatoTest Score Decrease	NashTest Score Decrease	AshTest Score Decrease
Non-T2DM	Median	0.13 (17.57%)	0.41 (66.13%)	0.09 (15.71%)	0.00 (0%)	0.01 (50.00%)
	IQ range	0.04–0.27	0.20–0.50	0.02–0.17	−0.17–0.14	−0.01–0.04
T2DM	Median	0.16 (22.54%)	0.22 (31.43%)	0.04 (7.14%)	0.00 (0%)	0.00 (0%)
	IQ range	0.10–0.25	0.08–0.51	−0.04–0.20	0.00–0.25	−0.03–0.01
***p* value**		**0.413**	**0.272**	**0.441**	**0.082**	0.009 *

* *p* < 0.05 when comparing the non-T2DM group with the TD2M group; IQ range: interquartile range.

**Table 4:** Fasting glucose levels before and after DAA treatment in non-T2DM patients vs. in T2DM patients.

Parameter	Statistic	Glucose Level (mg/dL)		
		Initial	Final	Decrease Level
Non-T2DM	Median	105.5	101.0	2.0
	IQ range	96.5–113.0	93.0–109.0	−9–11.0
T2DM	Median	176.0 *	137.0 *	27.0 *
	IQ range	141.0–264.0	110.0–239.0	106.0–7.0

Note: * *p* < 0.05 T2DM group compared with the non-T2DM group.

**Table 5:** Multivariate regression analysis of factors impacting the final fibrosis score.

Variable	FibroTest	ActiTest	SteatoTest	NashTest	AshTest
**Baseline value**	0.893 ± 0.082 *p* < 0.001 *	0.682 ± 0.120 *p* < 0.001 *	0.675 ± 0.112 *p* < 0.001 *	0.250 ± 0.189 *p* = 0.190	0.134 ± 0.048 *p* = 0.007 *
**Diabetes, type 2**	−0.013 ± 0.027 *p* = 0.641	0.054 ± 0.047 *p* = 0.261	0.011 ± 0.034 *p* = 0.747	−0.146 ± 0.079 *p* = 0.068	0.014 ± 0.008 *p* = 0.088
**Treatment-naive patient**	−0.024 ± 0.034 *p* = 0.475	−0.086 ± 0.060 *p* = 0.156	−0.009 ± 0.041 *p* = 0.025 *	0.007 ± 0.100 *p* = 0.946	0.001 ± 0.010 *p* = 0.980
**BMI**	0.015 ± 0.004 *p* < 0.001 *	0.028 ± 0.006 *p* < 0.001 *	0.010 ± 0.005 *p* = 0.027 *	0.015 ± 0.001 *p* = 0.142	0.001 ± 0.001 *p* = 0.129

Note: * *p* < 0.05.

## Data Availability

The dataset presented in this study is available from the corresponding author upon reasonable request.
